# Human papillomavirus in oral cavity and oropharynx carcinomas in the central region of Brazil^☆^

**DOI:** 10.1016/j.bjorl.2016.01.004

**Published:** 2016-04-09

**Authors:** Guilherme Petito, Megmar Aparecida dos Santos Carneiro, Sílvia Helena de Rabello Santos, Antonio Marcio Teodoro Cordeiro Silva, Rita de Cassia Alencar, Antonio Paulo Gontijo, Vera Aparecida Saddi

**Affiliations:** a Pontifícia Universidade Católica de Goiás (PUC-Goiás), Programa de Mestrado em Genética, Goiânia, GO, Brazil; b Instituto de Patologia Tropical e Saúde Pública, Universidade Federal de Goiás (UFG), Goiânia, GO, Brazil; c Associação de Combate ao Câncer em Goiás, Setor de Anatomia Patológica, Laboratório de Oncogenética e Radiobiologia, Goiânia, GO, Brazil

**Keywords:** Papillomaviridae, Papillomaviridae 16, Head and neck neoplasm, Epidemiology, Papillomaviridae, Papillomaviridae 16, Neoplasia de cabeça e pescoço, Epidemiologia

## Abstract

**Introduction:**

Molecular studies about carcinomas of the oral cavity and oropharynx demonstrate the presence of human papilomavirus genome in these tumors, reinforcing the participation of human papilomavirus in oral carcinogenesis.

**Objectives:**

This study aimed to determine the prevalence of human papilomavirus and genotype distribution of HPV16 and HPV18 in oral cavity and oropharynx carcinomas, as well as their association with clinical characteristics of the tumors.

**Methods:**

This is a retrospective study, with clinical data collected from 82 patients. Human papilomavirus detection was conducted on specimens of oral cavity and oropharynx carcinomas included in paraffin blocks. Patients were assisted in a cancer reference center, in the central region of Brazil, between 2005 and 2007. Polymerase chain reaction was used for the detection and genotyping of human papilomavirus.

**Results:**

Among the patients evaluated, 78% were male. The average age of the group was about 58 years. Risk factors, such as smoking (78%) and alcohol consumption (70.8%) were recorded for the group. HPV DNA was detected in 21 cases (25.6%; 95% confidence interval 16.9–36.6) of which 33.3% were HPV16 and 14.3% were HPV18. The presence of lymph node metastases and registered deaths were less frequent in human papilomavirus positive tumors, suggesting a better prognosis for these cases; however, the differences between the groups were not statistically significant.

**Conclusion:**

The results obtained in the present study, with respect to the presence of the high-risk HPV16 and HPV18 genotypes, highlight the importance of human papilomavirus vaccination in the control of oral cavity and oropharynx carcinomas.

## Resumo

**Introdução:**

Estudos moleculares sobre carcinomas da cavidade oral e orofaringe demonstram a presença do genoma do papilomavírus humano (HPV) nesses tumores, o que enfatiza a participação do HPV na carcinogênese oral.

**Objetivos:**

Esse Este estudo teve por objetivo determinar a prevalência de HPV e a distribuição genotípica de HPV16 e HPV18 nos carcinomas de cavidade oral e orofaringe, bem como sua associação com as características clínicas dos tumores.

**Método:**

Este é um estudo retrospectivo, com dados clínicos coletados de 82 pacientes. A detecção de HPV foi realizada em amostras de carcinomas de cavidade oral e orofaringe incluídos em blocos de parafina. Os pacientes foram atendidos em um centro de referência para tratamento do câncer, na região central do Brasil, entre 2005 e 2007. Foi utilizada a reação em cadeia de polimerase (PCR) para a detecção e genotipagem do HPV.

**Resultados:**

Entre os pacientes avaliados, 78% eram homens. A média de idade do grupo era de 58 anos. Fatores de risco como o tabagismo (78%) e consumo de álcool (70.,8%) foram registrados para o grupo. HPV DNA foi detectado em 21 casos (25,6%; IC de 95%, 16,9–-36,6), dos quais 33,3% eram HPV16 e 14,3% eram HPV18. A presença de metástases em linfonodos e os óbitos registrados foram menos frequentes em tumores positivos para HPV, sugerindo melhor prognóstico para esses casos; contudo, as diferenças entre os grupos não foram estatisticamente significantes.

**Conclusão:**

Os resultados obtidos no presente estudo, com respeito à presença de genótipos de alto risco de HPV16 e HPV18, destacam a importância da vacinação para HPV no controle dos carcinomas de cavidade oral e orofaringe.

## Introduction

Head and neck cancers (HNC), including oral cavity and oropharynx carcinomas, are the sixth most common cancer types in the world, with an annual estimate of 633,000 new cases and 355,000 deaths.^1^ In Brazil, 15,290 HNCs were expected in 2014, with 11,280 cases in men and 4010 in women.^2^

Oral and oropharynx carcinomas account for more than 80% of the total HNC cases,^3^ and squamous cell carcinoma (SCC) is the most common histological type, comprising more than 90% of the cases. The prognosis of these tumors is mostly pessimistic, with a low five-year survival of approximately 58%.^4^, ^5^

Age, gender, and tumor-node-metastasis (TNM) tumor staging, which includes the extension of the tumor, the presence of lymph node metastases, and distant metastasis, are the main prognostic factors for oral cavity and oropharynx carcinomas.^6^ In addition, histological grade and the expression of molecular markers (p16, pRb, and Ki-67) allow a better understanding of tumor behavior and evolution.^6^, ^7^, ^8^

The treatment of SCC of the oral cavity and oropharynx is usually accomplished by surgery or radiotherapy, alone or associated, and may also include the use of chemotherapy as an alternative to improve the chances of cure.^9^ Studies suggest that for Human papillomavirus (HPV)-positive oral cavity and oropharynx SCC, treatment with surgery and adjuvant radiotherapy is as good as the definitive radiotherapy treatment, with or without chemotherapy.^9^, ^10^

Smoking and alcohol consumption are considered the main risk factors for oral cavity and oropharynx cancer.^9^, ^10^, ^11^ However, with the intensification of campaigns against smoking and alcohol, the role of HPV in oral cavity and oropharynx carcinomas has gained prominence in recent years.^11^, ^12^ A growing number of studies support the hypothesis of HPV association with oral cavity and oropharynx carcinomas.^10^, ^11^, ^12^, ^13^, ^14^

HPV is a sexually-transmitted infection; therefore, factors such as early initiation of sexual activity, high number of sex partners, and the practice of unprotected oral sex are included as risk factors for HPV infection in the oral cavity and oropharynx mucosa.^15^ Growing incidence of oral cavity and oropharynx carcinomas associated with HPV in young people has been demonstrated.^14^, ^15^, ^16^

The prevalence of HPV in oral cavity and oropharynx SCC is the focus of several studies worldwide; HPV16 is considered the most prevalent and relevant genotype in the epidemiology of these carcinomas.^13^, ^17^, ^18^, ^19^, ^20^

This study aimed to investigate the prevalence and genotypic distribution of HPV16 and HPV18 in oral cavity and oropharynx carcinomas, as well as their possible association with the clinical characteristics of the tumors.

## Methods

### Type of study and series

The study was approved by the Research Ethics Committee, under No. 13580613.5.0000.0031/2014. It was a retrospective cross-sectional study that used data collected from medical files and analysis of paraffin blocks containing specimens of oral cavity and oropharynx carcinomas. The selection was first accomplished by analyzing the records of the Pathology Department at the Hospital, and only patients with histological diagnosis of SCC of oral cavity and oropharynx were included. Initially 312 cases of SCC diagnosed in the period from 2005 to 2007 were selected. After eliminating duplicates, 174 cases with available clinical records were selected. Patients who received chemotherapy/radiotherapy before surgery were excluded, resulting in 108 cases. Paraffin blocks from 108 cases were histologically examined, and specimens with exiguous amount of tumors were excluded, resulting in 82 cases that were selected for DNA extraction and HPV DNA detection.

### DNA extraction

Genomic DNA was purified from tumor samples fixed in formalin and included in paraffin. The samples were dewaxed in xylene and washed in ethanol according to standardized protocol. DNA was isolated by using the commercial Wizard Kit (Promega). The presence and the integrity of the DNA were verified by the amplification of a 99 base pair (BP) fragment from glyceraldehyde-3-phosphate dehydrogenase (GAPDH), by using polymerase chain reaction (PCR).

### HPV DNA detection

HPV DNA was detected by PCR. The set of primers used was SPF 1/2 (short PCR fragment). The SPF 1/2 amplifies a fragment of 65 pb of the L1 region of HPV genome. This set of primers allow the detection of 39 high and low oncogenic risk HPV: 6, 11, 13, 18, 26, 30, 31, 33, 34, 35, 39, 40, 42, 43, 44, 45, 51, 52, 53, 54, 55, 56, 58, 59, 61, 62, 64, 66, 67, 68, 69, 70, 72, MM4, MM7, 73, 74, and MM8. The genotypes detected by SPF 1/2 are those that infect the mucous membrane.^13^ PCR with SPF 1/2 primers was carried out in a final reaction volume of 25 μL, adding 2 μL of purified DNA, 2.5 mM/L MgCl_2_, 2 mM/L of each deoxyribonucleotide (dNTPs), 2.5 μM/L of each oligonucleotide primer, 0.25 U Taq polymerase (Invitrogen, Brazil) and ultra pure water in sufficient quantity for the final volume. Cycling conditions included: preheating for 1 min at 94 °C, followed by 40 cycles of: 94 °C for 1 min, 1 min at 45 °C, and 1 min at 72 °C, with a final extension of 5 min at 72 °C. Positive and negative controls were used in each reaction.

### Genotyping of HPV16 and 18

HPV16 and HPV18 genotyping was performed by PCR for all HPV-positive tumors. Two sets of primers that amplify part of E7 region of each HPV genome were employed.^21^ For the HPV16 genome, the amplicon presents 108 pb, and for HPV18 genome amplification, the amplicon presents 104 pb. PCR with HPV16 primers was performed in a final reaction volume of 25 μL, with 2 μL of purified DNA, 2.5 mM/L MgCl_2_, 2 mM/L of each dNTPs, 2.5 μM/L of each oligonucleotide primer, 0.25 U Taq polymerase (Invitrogen, Brazil), and ultra pure water in sufficient quantity for the final volume. Cycling conditions included: preheating for 1 min at 94 °C, followed by 40 cycles of: 94 °C for 1 min, 1 min at 45 °C, and 1 min at 72 °C, with a final extension of 5 min at 72 °C. PCR with HPV18 primers was performed in a final reaction volume of 25 μL, with 5 μL of purified DNA, 2.5 mM/L MgCl_2_, 2 mM/L of each dNTPs, 2.5 μM/L of each oligonucleotide primer, 1.25 U Taq polymerase, (Invitrogen, Brazil) and ultra pure water in sufficient quantity for the final volume. Cycling conditions included: preheat for 3 min at 94 °C, followed by 35 cycles of: 1 min at 94 °C, 1 min at 53 °C, and 1 min at 72 °C, with a final extension of 3 min at 72 °C.

### Statistical analysis

All the patients’ data were transcribed to Microsoft Excel^®^ spreadsheets. Clinical and histological data of the group, as well as the presence of HPV genome, HPV16, and HPV18 genotypes were analyzed by using Fisher's exact test and the chi-squared test. Values of *p* ≤ 0.05 were considered statistically significant. Positive and negative controls were used in each reaction.

## Results

Clinical and histological characteristics: a group of 82 cases of SCC of oral cavity and oropharynx were selected and evaluated with respect to clinical and histological characteristics (Table 1). Among the patients, 78% were male. Most of the patients (54.9%) were in the age group under 59 years and 62.2% were married. Risk factors, such as smoking (78%) and alcohol consumption (70.8%) were recorded for the majority of the group. Data associated with sexual behavior and orientation, such as the number of sexual partners, age at sexual activity onset, and practice of oral sex were not reported in the medical files.

**Table 1:** Analysis of the clinical and histological characteristics of the patients with oral cavity and oropharynx carcinomas.

Variable	All cases (*n* = 82)	
	*n*	%
*Gender*		
Female	18	22.0
Male	64	78.0

*Age at diagnosis*		
Average	58	–
≤59	45	54.9
≥60	37	45.1

*Marital status*		
Single	15	18.2
Married	51	62.2
Other	16	19.6

*Smoking*		
Yes	64	78.0
No	18	22.0

*Alcohol consumption*		
Yes	58	70.8
No	24	22.0

*Tumor location*		
Oral cavity	39	47.6
Oropharynx	43	52.4

*Staging*		
I/II	14	17.1
III/IV	68	82.9

*Tumor size*		
T1 and T2	35	42.7
T3 and T4	47	57.2

*Lymph node metastasis*		
Yes	42	51.2
No	40	48.8

*Distant metastases*		
Yes	01	1.2
No	81	98.8

*Histological grade*		
Low	13	15.8
Moderate/high	69	84.2

*Registered death*		
Yes	28	34.1
No	54	65.9

HPV detection and genotyping: HPV DNA was detected in 21 cases (25.6%; 95% confidence interval (CI) 16.9–36.6), of which 33.3% were HPV16 and 14.3% were HPV18. Table 2 describes the clinical characteristics of HPV-positive and negative cases. Among the 21 HPV-positive samples, 47.4% were located in the oral cavity and 52.6% in oropharynx. Considering the clinical staging for HPV-positive tumors, 4.8% were in stages I/II, while 95.2% were in stages III/IV. Lymph node metastases were detected in 42.9% of HPV-positive cases and in 57.4% of HPV-negative cases (*p* = 0.08). Distant metastases were not detected in HPV-positive cases, while one distant metastasis was described in the HPV-negative cases (*p* = 0.46). With respect to histological grade, 85.7% of HPV-positive cases showed moderate to high grade of differentiation. A greater number of deaths were registered in the HPV-negative group (39.3%) compared to the HPV-positive group (19.1%), though this difference was not statistically significant (*p* = 0.11). With respect to HPV genotyping, seven samples (33.3%) were HPV16 positive, while three samples (14.3%) were HPV18 positive. Significant differences between HPV16 and HPV18 tumors were not detected in this study.

**Table 2:** Analysis of the clinical and histological characteristics of the patients with HPV-positive and HPV-negative oral cavity and oropharynx carcinomas.

Variable	HPV (−) (*n* = 61)		HPV (+) (*n* = 21)		*p*^a^
	*n*	%	*n*	%	
*Gender*					
Female	15	24.6	03	14.3	0.38
Male	46	75.4	18	85.7	

*Age at diagnosis*					
Average	60	–	53	–	0.31
≤59	31	50.8	14	66.7	
≥60	30	49.2	07	33.3	

*Tumor location*					
Oral cavity	29	47.5	10	47.4	1.00
Oropharynx	32	54.5	11	52.6	

*Staging*					
I and II	13	21.3	1	4.8	0.10
III and IV	48	78.7	20	95.2	

*Tumor size*					
T1 and T2	26	42.6	09	33.3	1.00
T3 and T4	35	57.4	12	66.7	

*Lymph node metastasis*					
Yes	35	57.4	07	42.9	0.08
No	26	42.6	14	57.1	

*Remote metastases*					
Yes	01	1.6	00	0.00	0.46
No	60	98.4	21	100.0	

*Grade of differentiation*					
Low	10	16.4	03	14.3	1.00
Moderate, high	51	83.6	18	85.7	

*Registered deaths*					
Yes	24	39.3	04	19.1	0.11
No	37	60.6	17	80.9	

*Treatment*					
Surgery only	24	39.5	03	14.3	0.32
Surgery + radiotherapy	14	22.9	08	38.1	
Radiotherapy + chemotherapy	09	14.7	03	14.3	
Other	14	22.9	07	33.3	

*HPV type*					
HPV16	–	–	07	33.3	–
HPV18	–	–	03	14.3	
Other	–	–	11	42.4	

a All *p*-values were calculated with Fischer's exact test.

## Discussion

In this study analyzing 82 cases of SCC of oral cavity and oropharynx diagnosed in a cancer reference center in the central region of Brazil. HPV DNA was detected in 21 cases (25.6%), of which 33.3% were HPV16 and 14.3% were HPV18. These results support the hypothesis that a subgroup of oral cavity and oropharynx carcinomas is HPV related.^12^, ^13^, ^14^, ^15^, ^16^, ^17^, ^18^ Significant changes in the epidemiology of mucosal SCCs of the head and neck, with an increasing number of cases related to HPV, have been demonstrated in the last decade.^1^, ^2^, ^3^, ^4^, ^5^, ^6^, ^7^, ^8^, ^9^, ^10^, ^11^, ^12^, ^13^, ^14^, ^15^, ^16^, ^17^, ^18^, ^19^, ^20^, ^21^, ^22^ In addition to tobacco and alcohol consumption, HPV appears as an important risk factor for oral cavity and oropharynx SCC development. The prevalence of HPV DNA in oropharyngeal cancer (OC) varies in different studies, and up to 84% of cases have been associated to HPV infection.^19^

The association between HPV infection with oral cavity and oropharynx SCC emphasizes the importance of introducing specific molecular tests into oral cancer prevention practices, in order to evaluate the presence of the virus and the possibility of expanding anti-HPV vaccine in the male population.^23^, ^24^

In the present study, 54.9% of the patients were younger than 59 years. Several studies describe the age range of the patients with carcinoma of oral cavity and oropharynx as similar to the value described in the present study.^25^, ^26^ In a study conducted in Italy, the median age was 65.6 years.^18^ The accumulation of exposure to various risk factors, such as lifelong smoking and alcoholism, contributes to the higher prevalence of these tumors in more advanced ages.^6^ Studies demonstrated that HPV-associated HNC, including oropharyngeal and oral cavity SCCs, have recently risen dramatically in men under 50 years old.^27^ In a period of 20 years, the relative prevalence of HPV-positive oropharyngeal squamous cancer cell (OSCC) went from less than 20% to more than 70% in the United States and some European countries.^28^, ^29^, ^30^ In the HPV-positive patients evaluated in the present series, 66.7% of the cases were under the average age (59 years). Most cases of carcinoma of the oral cavity and oropharynx were observed in males (78.0). Various authors have previously reported this information; however, in different countries of Europe, an increasing tendency in the incidence of oropharynx carcinomas has been noticed in females.^9^, ^10^, ^11^, ^12^, ^13^, ^14^, ^15^, ^16^, ^17^, ^18^, ^19^, ^20^, ^21^, ^22^, ^23^, ^24^, ^25^, ^26^, ^27^, ^28^, ^29^

Smoking and alcohol consumption are described as the main risk factors for carcinomas of the oral cavity and oropharynx.^11^, ^12^, ^13^, ^14^, ^15^, ^16^, ^17^, ^18^, ^19^, ^20^, ^21^, ^22^, ^23^, ^24^, ^25^, ^26^, ^27^, ^28^, ^29^, ^30^, ^31^ The present study confirmed the high frequency of smokers (78.0%) and alcohol drinkers (70.8%) in the group. Although not fully considered as a prognostic factor, carcinomas of the oral cavity and oropharynx associated with smoking and alcoholism tend to be more aggressive.^1^ Tobacco and alcohol can induce SCC of the oral cavity and oropharynx by aggression on extensive areas, leading to a phenomenon known as field cancerization, which is characterized by molecular changes in the reserve cells, leading to the formation of epithelial field changes.^26^ This field undergoes expansion and extends over the surface of the mucous membrane, increasing the possibility of formation of a new carcinoma.^32^, ^33^

The profile of patients with carcinoma of the oral cavity and oropharynx associated with HPV tends to be characterized by a group of younger patients, aged less than 60 years, non-smokers or light smokers, non-drinkers, and with more promiscuous sexual behavior; however, these data are controversial.^34^, ^35^ One important limitation of the present study is the lack of data related to the patients’ sexual behavior. Since it was a retrospective study, such data were not available in the medical files. Although a higher proportion of patients were described under 59 years in the HPV-positive group (66.7%) compared to the HPV-negative group (50.8%), these differences were not statistically significant. Similar results with respect to age group were described in two studies developed in the United States.^13^, ^14^ In the present study, the average age for HPV-positive patients was lower, 53 years, compared to the HPV-negative group, 60 years. A study conducted in Colombia confirmed a lower average age for HPV-positive patients with oral cavity and oropharynx carcinomas.^12^ Significant associations between the presence of HPV and the absence of habits like smoking and alcohol consumption were not noticed in the present study, corroborating with various studies.^6^, ^7^, ^8^, ^9^, ^10^, ^11^, ^12^, ^13^, ^14^, ^15^, ^16^, ^17^, ^18^

The prognosis of oral cavity and oropharynx carcinomas is uncertain and difficult to predict.^4^ The identification of factors that may help choosing the best treatment, predicting the evolution of the tumor as well as patient's survival, is of evident clinical importance. HPV-positive oral cavity and oropharynx SCC seem to present a better prognosis when treated with surgery and adjuvant radiotherapy or radiation therapy with or without final chemotherapy.^10^ Limitations on data collection in the present study prevented a survival analysis associated with the therapy used. A systematic review highlights the importance of evaluating patients with HPV-related oropharyngeal carcinomas, in order to predict the best therapy, since these virus-associated carcinomas show distinct molecular characteristics compared to HPV-negative ones.^9^ However, currently, only a few studies describe a direct association between treatment, the presence of the HPV genome, and survival.^9^, ^10^

A better prognosis for HPV-positive oral cavity and oropharynx carcinomas has been described in a few studies, including well-differentiated tumors, with less risk of lymph node metastasis and distant metastasis.^9^, ^10^, ^11^, ^12^, ^13^, ^14^, ^15^, ^16^, ^17^, ^18^, ^19^, ^20^, ^21^, ^22^, ^23^, ^24^, ^25^, ^26^, ^27^, ^28^, ^29^, ^30^, ^31^, ^32^, ^33^, ^34^, ^35^, ^36^ In the present study, these characteristics were observed; however, the difference between the groups was not significant. Distant metastases were not described in the HPV-positive group, although only one case of HPV-negative tumors presented with distant metastases. Concerning the presence of lymph node metastasis, a lower rate of lymph node metastasis was observed in the HPV-positive tumors (42.9% *vs*. 57.4%), suggesting a better prognosis for these tumors; however, such differences were not statistically significant.

HPV16 and HPV18 are described as the most prevalent genotypes in oral cavity and oropharynx carcinomas, and their association with these carcinomas seems to be relevant.^14^ In the present study, of the 21 HPV-positive samples, HPV16 was described in 33.3% and HPV18 in 14.3% of the tumors. To describe the prevalence of HPV16 and 18 in oral cavity and oropharynx carcinomas is important in order to predict the impact of vaccination on these tumors, since both genotypes are the main targets for the bivalent (16/18) and quadrivalent vaccines (11/16/18/6).^15^, ^16^, ^17^, ^18^, ^19^, ^20^, ^21^, ^22^, ^23^, ^24^, ^25^, ^26^, ^27^ In the present study, no significant differences were described regarding the staging and death of the patients when comparing HPV16 and 18 positive and negative cases.

Recent reports highlight that vaccination campaigns are an efficient solution for the control of the HPV-associated carcinomas.^15^, ^16^, ^17^, ^18^, ^19^, ^20^, ^21^, ^22^, ^23^, ^24^, ^25^, ^26^, ^27^, ^28^, ^29^, ^30^, ^31^, ^32^, ^33^, ^34^, ^35^, ^36^, ^37^ Regarding this connection and considering the high prevalence of HPV in oral cavity and oropharynx carcinomas in males, it is extremely important that this campaign might also be extended to the male group.

In the present study, the presence of HPV was detected in 25.6% of the cases, including types 16 and 18, leading to the conclusion that HPV is also associated with the development of oral cavity and oropharyngeal carcinomas. This study presented an important limitation with respect to clinical and behavioral data of the patients. In the medical files, such data were scarce, including sexual behavior, oral hygiene, history of sexually transmitted diseases, and patient follow-up, among others. These difficulties are inherent to retrospective studies, since the researchers rely only on the information present in medical records that can often be lost, incomplete, or unclear.^20^, ^21^, ^22^, ^23^, ^24^, ^25^, ^26^, ^27^, ^28^, ^29^, ^30^

The authors suggest a prospective study, with more efficient data collection and patient follow-up, allowing a more accurate and complete survey, with a larger number of cases from different oncology centers in the country, in order to improve the knowledge of HPV carcinogenesis in oral cavity and oropharynx carcinomas.

## Conclusions

This study confirms the higher prevalence of HPV DNA in oral cavity and oropharynx carcinomas, especially in males (78%), with an average age of 58 years, and a high frequency of smokers and alcohol drinkers.

The prevalence of HPV DNA genome in the samples analyzed was 25.6%, and among the positive cases, 33.3% were HPV16 and 14.3% were HPV18, highlighting the association of high-risk HPV in oral cavity and oropharynx carcinogenesis.

## Conflicts of interest

The authors declare no conflicts of interest.
